# A coil in the hair—a case report of percutaneous coil migration

**DOI:** 10.1007/s00701-018-3689-3

**Published:** 2018-10-03

**Authors:** Bart De Boer, Alfred P. See, H. Bart Brouwers, Gabriël Rinkel, Ciro Princiotta, Marike L. D. Broekman

**Affiliations:** 1 Department of Neurosurgery, Brain Center Rudolf Magnus, University Medical Center Utrecht, HP G03.124, PO Box 85500, 3508 GA Utrecht, The Netherlands; 2 Department of Neurosurgery, Brigham and Women’s Hospital, Boston, MA 02115 USA; 3 Department of Radiology, Instituto delle Scienze Neurologiche di Bologna, Ospedale Bellaria, Bologna, Italy

**Keywords:** Foreign body migration, Therapeutic embolization, Craniotomy, Intracranial arteriovenous malformations, Cerebral hemorrhage

## Abstract

Coil migration following cerebral aneurysm treatment has been described and may result in stroke, recurrent aneurysm, or local mass effect. Cerebral coil embolization is also applied in arteriovenous malformations and arteriovenous fistulas, but these pathologies are relatively rare and coil migration is not as well described. Furthermore, these cases are more commonly treated with combinations of multiple modalities to achieve cure. Embolization, surgery, and radiation each have risks and benefits and combinations may have synergistic risks and benefits not seen in monotherapy. We report a case of extravascular and extra-corporeal coil migration after embolization and craniectomy to treat a patient with hemorrhage from an arteriovenous fistula.

**Electronic supplementary material:**

The online version of this article (10.1007/s00701-018-3689-3) contains supplementary material, which is available to authorized users.

## Background and importance

Even though hybrid open cerebrovascular and neuroendovascular approaches have been performed for many years, recently, they are increasingly applied to treat challenging pathology as more medical centers install hybrid operating-angiography suites. In a positive feedback loop, increasing experience with the hybrid environment has allowed more aggressive treatment of complex cerebrovascular disease which previously may have been considered incurable [1, 2]. However, the complications of hybrid approaches are not as well understood and may be distinct from the complications of each technique in isolation. Intracranial coil embolization via a femoral arterial approach is not known to have a risk of trans-cutaneous migration. This case introduces the concept of unforeseen complications due to new hybrid approaches.

## Clinical presentation/case report

**Fig. 1:**
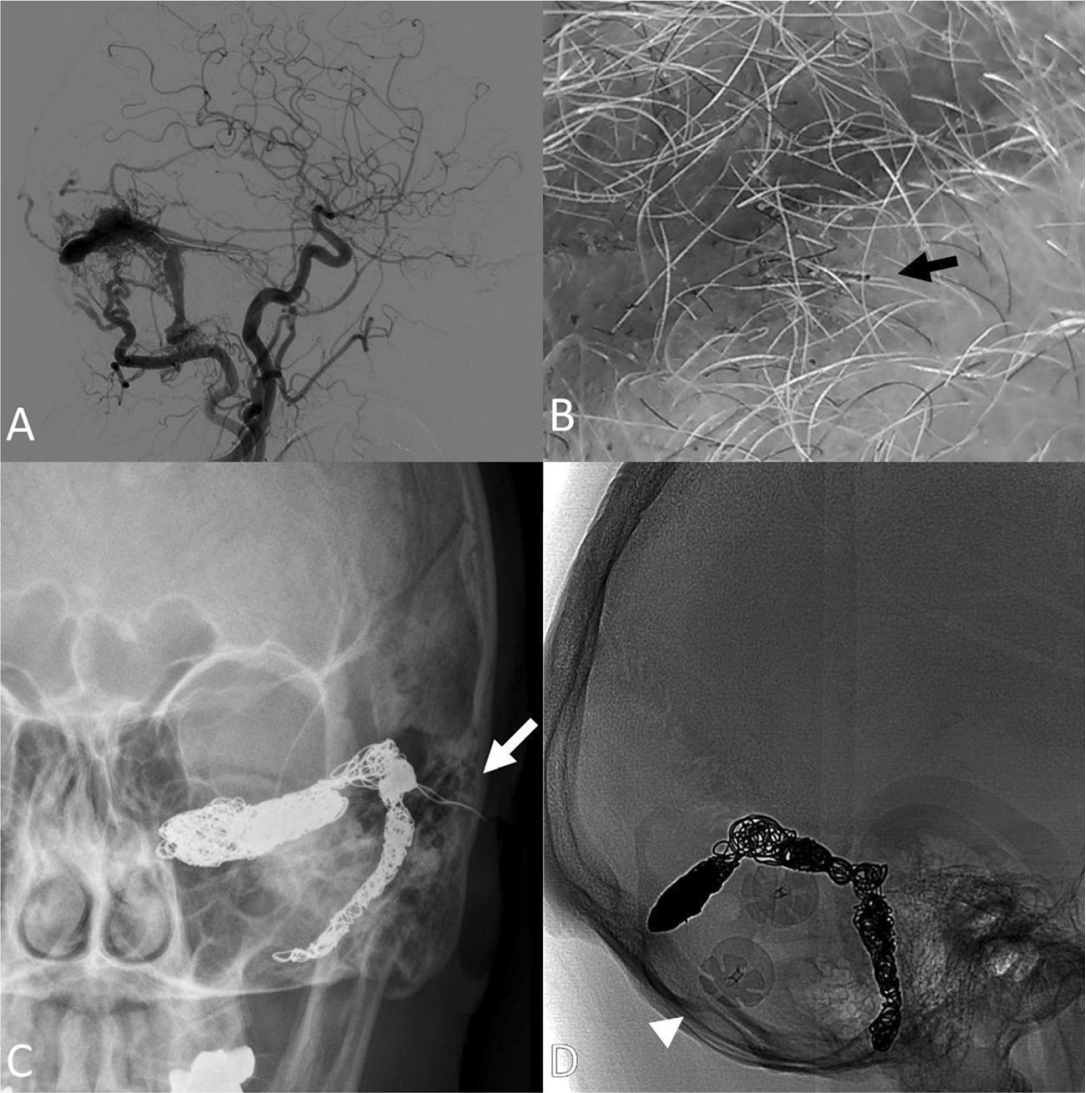
**a** Lateral projection digital subtraction angiography demonstrating the arteriovenous fistula with shunting from the left occipital artery and left medial tentorial artery, and with early cortical reflux. **b** Photograph demonstrating the protruding coil (black arrow). **c** Anterior-posterior projection x-ray of the skull demonstrating the coil embolization mass with protrusion of the coil extracranially through the craniectomy defect (white arrow). **d** Lateral projection native fluoroscopic image after craniotomy for resection of migrated coil (absent coil strand along the embolization-access tract) with reconstructive plates (arrowhead)

During an international trip, a 60-year-old man experienced left temporal-occipital hemorrhage and was found to have an arteriovenous fistula (AVF, Fig. 1a) with an isolated sinus and retrograde cortical venous drainage without feasible endovascular trans-venous access, which was tried several times. Instead, embolization was performed with trans-cranial direct puncture of the AVF with a 16-gauge angiocatheter under fluoroscopic guidance. At the end of the procedure, at time of extracting the angiocatheter, the coil began to come out of the sinus (Supplemental Fig. 1). The boneflap was not placed back because of edema associated with the hemorrhage. The coil did not cross the skin. Several weeks after returning home, 6 weeks after surgery, he followed up at University Medical Center (UMC) and reported excellent neurologic recovery, but inquired about a sharp protrusion through the scalp on the site of the operation. He had noticed it while washing his hair 1 week before, and his wife had trimmed this with scissors. He did not notice any other associated symptoms such as fever, local pain, erythema, drainage, or swelling. On physical examination, he was alert and oriented, with complete cranial nerve function and appropriate strength and sensation. On examination of his scalp, there appear to be a metallic foreign body consistent with coil protruding from the skin (Fig. 1b).

The patient received flucloxacillin, a standard first-line antibiotic agent at UMC, and taken to the operating room for resection and washout. Dissection exposed the subgaleal-epidural space. Intradural exploration was not pursued, but the coil was transected at the dural surface (Fig. 1d). An acrylic cranioplasty flap was applied. The patient recovered well from this procedure and had no further complications. Follow-up of the arteriovenous fistula demonstrated no further arteriovenous shunting or venous congestion.

## Discussion

Migration is a known complication of coil embolization. In particular, intravascular migration into an unintended vessel is frequently observed intraprocedurally and may result in incomplete embolization or off target embolization, occurring in approximately 3% of cases [3]. The subarachnoid space is a second location for coil migration and may be the underlying cause of long-term recanalization in as many as 55% of embolized aneurysms [4]. Extravascular coil migration may lead to recurrent risk of aneurysm rupture and local mass effect on cranial nerves [5]. However, extra-corporeal migration of coils is not a known complication of intracranial coil embolization. This case represents a novel complication unique to the evolving field of hybrid open-endovascular treatments.

Coil embolization is also applied in the systemic endovascular therapy for pseudoaneurysms. In the superficial coil embolization with thin overlying soft tissue, such as at the posterior tibial artery, coil migration may develop a cutaneous wound [6]. However, due to the calvarium, neuroendovascular coil embolization in general is not susceptible to this complication. In the presented case, the combination of a superficial convexity embolization target and the craniectomy minimized the barriers to cutaneous migration. In the setting of craniectomy, cerebral pulsations and cerebral spinal fluid flow are also altered, which may have increased a propensity for coil migration.

Some find the trans-venous approaches safer than trans-arterial approaches in the treatment of this type of AVF. However, specific to the trans-cranial trans-venous approach sometimes, in the final stages of embolization, when the sinus is largely occupied by the coils, the angiocatheter, that is short and unstable, can be displaced out of the sinus with partial protrusion of the extremity of the last coil through the dural breach. In this case, the healing of the fistula is not compromised, but in the absence of bony coverage then over time the metallic material may progressively pass through the overlying soft tissue and expose the patient to the risk of infection.

Hybrid operating and angiography suites are increasingly common. As hybrid practitioners refine novel techniques, previously unforeseen challenges and complications are expected to arise [1]. Combined endovascular and surgical treatments for arteriovenous fistulas and malformations are indicated only when the combined risk-benefit profile is better than that of monotherapy. This complication is a unique result of combinatorial therapy. After embolization, during the open surgery, resection of embolic product such as coils or liquid embolic agents may eliminate this possibility. Early cranioplasty may also prevent this complication.

## Conclusion

The patient presented with a non-infected cutaneous wound from extravascular coil migration 6 weeks after hybrid open-endovascular approach to coil embolization of an AVF. Extradural coil mass was resected and a synthetic cranioplasty flap was applied. This case demonstrates a potential synergy of risks in hybrid open and endovascular therapies, resulting in new unforeseen complications.

## Electronic supplementary material

Supplemental Figure 1 Lateral (A) and Anterior-posterior (B) projection digital subtraction angiography showing the migration of the coil at the end of the combined procedure. (JPG 21 kb)
