# Supplementary figures and images for: A coil in the hair—a case report of percutaneous coil migration
Source: Acta Neurochir (Wien). 2018 Oct 3;160(12):2397–9. doi: 10.1007/s00701-018-3689-3 (PMC6267699; doi:10.1007/s00701-018-3689-3)

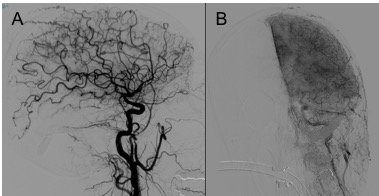

Supplement: Supplementary file 1 — Lateral (A) and Anterior-posterior (B) projection digital subtraction angiography showing the migration of the coil at the end of the combined procedure. (JPG 21 kb) [file 701_2018_3689_MOESM1_ESM.jpg]
